# Recent advances in understanding of enterobacterial common antigen synthesis and regulation

**DOI:** 10.1098/rsob.250055

**Published:** 2025-07-02

**Authors:** Haley C. Hill, Angela M. Mitchell

**Affiliations:** ^1^ Department of Biology, Texas A&M University, College Station, TX, USA

**Keywords:** enterobacterial common antigen, surface antigens, O-antigen, gene expression, permeability, outer membrane

## Abstract

Antibiotic resistance has become one of the most significant global health threats, with nearly three million antibiotic-resistant infections occurring per year in the United States alone. This problem is especially acute in Gram-negative bacteria, which possess an outer membrane (OM) that surrounds the aqueous periplasm and cytoplasmic membrane and acts as a permeability barrier capable of excluding many antibiotics. The OM of Enterobacterales also contains a highly conserved, invariant carbohydrate-derived moiety known as enterobacterial common antigen (ECA), which has been shown to play a significant role in this membrane permeability barrier. Although ECA was first discovered in the 1960s, its precise function and regulation remain largely mysterious. Here, we highlight recent studies that have advanced our understanding of the structure, biosynthesis, regulation and potential functions of ECA. We also review new insights into the complex interactions of the cell envelope biosynthesis pathways which may also play a role in surface antigen biosynthesis.

**Figure:**
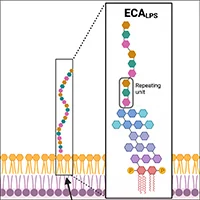

## 1. Introduction

**Figure 1.**
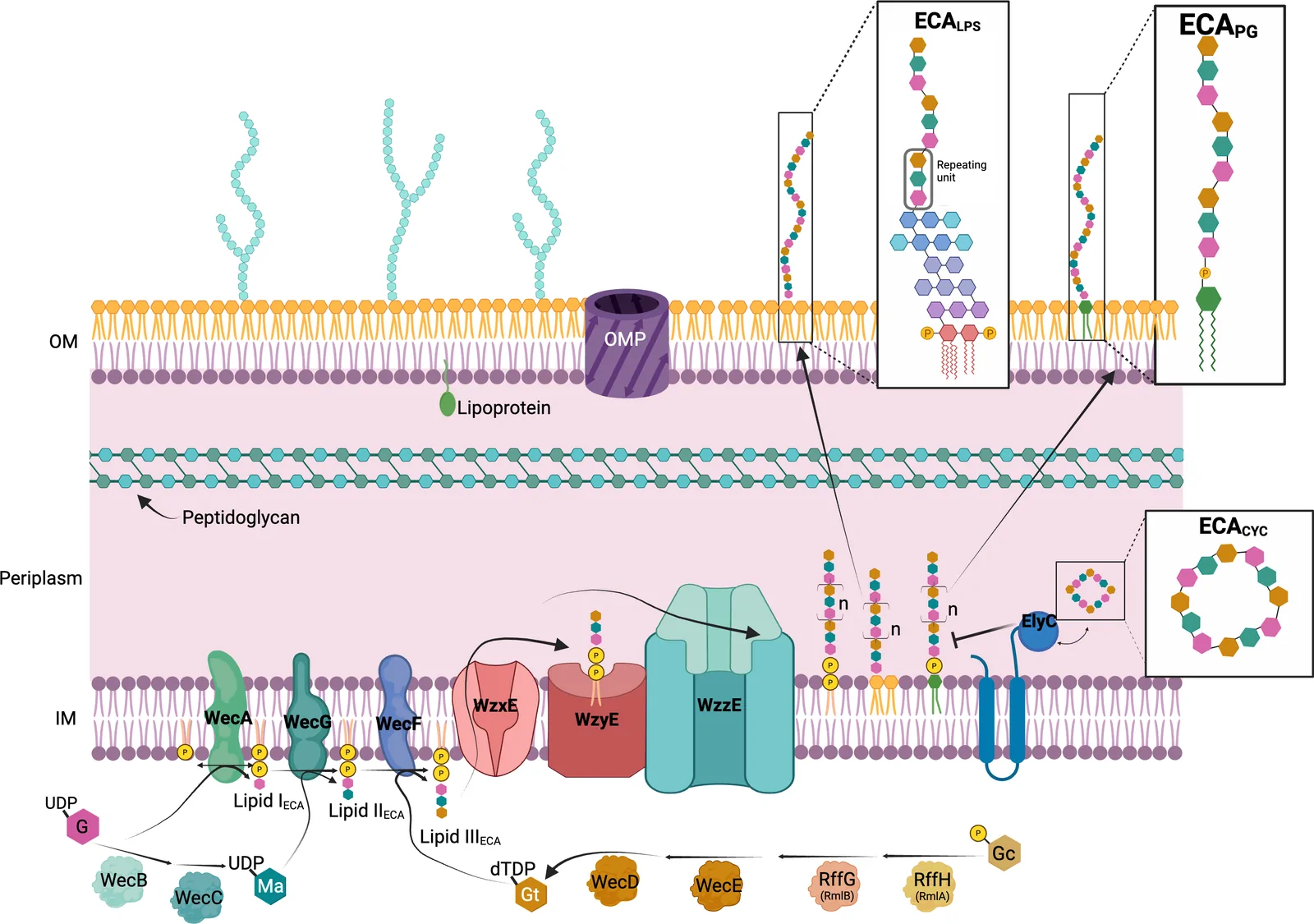
Schematic representation of ECA Biogenesis in *E. coli*. ECA biogenesis begins with synthesis of the three amino sugars: *N-*acetyl-d-glucosamine (G), *N-*acetyl-d-mannosaminuronic acid (Ma) and 4-acetamido-4,6-dideoxy-d-galactose (Gt). The amino sugars are then loaded sequentially onto the isoprenoid carrier (Und-P) by a series of transferases, dehydrogenases, epimerases, including WecA, WecB, WecC, WecD, WecE, WecF, WecG until it forms a complete ECA subunit. The isoprenoid-linked precursor is then flipped across the IM by the WzxE flippase, and the subunits are polymerized by WzyE which coordinates in a complex with the chain length regulator protein WzzE. The three forms of ECA made with these polymerized subunits are: the surface-exposed, linear ECA_PG_, which is transferred off the isoprenoid carrier and attached to phosphatidylglycerol via a phosphodiester linkage before being transferred to the OM via an, as yet, unknown mechanism; the surface-exposed, linear ECA_LPS_, which is transferred off the isoprenoid carrier and attached to LPS before being transferred to the OM via what is assumed to be the Lpt pathway; and the periplasmic ECA_CYC_, which is transferred off the isoprenoid carrier and retained in the periplasm by a yet unknown mechanism and works in coordination with the IM protein ElyC to regulate ECA_PG_ levels. Created in BioRender (Bennett, H. (2025) https://BioRender.com/u23n235).

The problem of antibiotic resistance is especially acute in Gram-negative bacteria, where the outer membrane (OM), which surrounds the aqueous periplasm, peptidoglycan cell wall and cytoplasmic membrane (see figure 1), acts as a permeability barrier capable of excluding many antibiotics [1–6]. Gram-negative bacteria, including species in the order Enterobacterales, are intrinsically more resistant to antibiotics than Gram-positive bacteria due to the presence of the OM [7–9]. The OM is an asymmetric bilayer composed of mainly lipopolysaccharides (LPS) in the outer leaflet and phospholipids in the inner leaflet [1,10,11]. Various lipoproteins are localized in the inner leaflet, and in some cases the outer leaflet of the OM [12,13]. Lipoproteins are involved in the LPS transport system (Lpt) [14,15], the retrograde phospholipid re-localization pathway (Mla) [16], outer membrane protein (OMP) folding (Bam) [17], the lipoprotein trafficking pathway (Lol) [18,19] and peptidoglycan synthesis [20,21]. OMPs span both leaflets and act as porins for small, hydrophilic molecules and, in some cases, lipoproteins [2,22]. OMPs, which form β barrels, also perform a wide range of functions including acting as adhesion factors in virulence [23], as well as channels for the uptake of nutrients [1], siderophore receptors [24] and enzymes [25]. These OM components play critical roles in keeping the permeability barrier function of the OM intact and preventing the passage of many harmful molecules. Moreover, OM permeability has been shown to decrease in responses to cellular stresses [26,27], thus there is an urgent need to study the function and biosynthesis of OM components.

In addition to its effect on cell permeability, the OM provides an additional layer of protection against extracellular stressors [28–32]. For example, LPS, proteins and O antigen in the OM contribute to its stiffness and serve to protect cells against osmotic pressure [3,4,33–35]. The presence of outer membrane vesicles (OMVs) is posited to protect against host defences by enabling bacterial survival during stress conditions and regulating bacterial community interaction [36]. Moreover, the OM provides high hydrophilicity, which is important in evading phagocytosis and host immune defences by changing the surface antigen composition [37].

The OM of Enterobacterales also contains a highly conserved invariant carbohydrate-derived moiety known as enterobacterial common antigen (ECA) [38], which plays a critical role in the membrane permeability barrier [1,35,39]. ECA has three forms: a cell surface linear polysaccharide linked to diacyl-glycerol phosphate, a.k.a. phosphoglyceride (ECA_PG_) [40], a cell surface linear polysaccharide linked to LPS (ECA_LPS_) [41] and a cyclic form (ECA_CYC_) [42] found in the periplasm (see figure 1). All three forms of ECA are made from repeating units of three amino sugars. Although ECA was first discovered in the 1960s, its specific function and regulation remain largely uncharacterized. Interestingly, a number of roles for ECA have been identified over the years, with ECA having importance for resistance to a vast assortment of external stressors such as bile salts (like those present small intestine), acetic acid (the most common short-chain fatty acid (SCFA) found in the human gut), certain antibiotics such as vancomycin, detergents such as benzalkonium chloride, and human serum (reviewed in [40]). Over the many years, significant effort has been poured into determining the structure, biosynthesis and regulation of ECA in an effort to clarify the decades-old mystery of ECA’s function in protecting Gram-negative enteric bacteria from this wide variety of external stressors, the current understandings of which will be discussed in the following sections.

## 2. Bacterial cell envelope structure and features

The cytoplasmic or inner membrane (IM), a phospholipid bilayer that surrounds the cytoplasm, is composed of phosphatidylethanolamine (PE), phosphatidylglycerol (PG) and cardiolipin (CL) in Enterobacterales [3]. PE comprises about 75% of the phospholipids with the remaining made up of PG (20%) and CL (5%) [43–45]; however, in the stationary phase, the amount of CL increases at the expense of PG [46]. IM phospholipids distribution is asymmetric with higher PE levels in the inner leaflet than the outer leaflet [29]. There is also variability in the lateral distribution of phospholipids in each leaflet [29,30], but it is not yet known what molecular mechanisms account for the phospholipid asymmetry.

The peptidoglycan cell wall protects against turgor pressure, defines cell shape, and provides a scaffold for anchoring other envelope components [31,32]. Peptidoglycan is composed of repeating *N*-acetyl glucosamine-*N*-acetyl muramic acid glycan strands crosslinked by peptide side chains [33]. In Gram-positive bacteria, the peptidoglycan cell wall is very thick (30–100 nm), compared to the Gram-negative peptidoglycan layer, which resides in the periplasm attached to the OM through protein interactions and is less than 5 nm thick [3].

The OM of Gram-negative bacteria is composed of LPS in its outer leaflet and phospholipids in its inner leaflet, with many lipoproteins and integral OMPs (e.g. porins) (see figure 1). OMPs are transmembrane β-barrel proteins and perform functions such as maintaining OM structure, host cell adhesion, invasion and anchoring, as well as LPS modification and metabolite transport [47–50]. OMP porins allow small hydrophilic molecules across the membrane including water, ions and nutrients. Porin OMPs such as OmpF also allow small hydrophilic antibiotics like β-lactams, tetracyclines, chloramphenicol and fluoroquinolones to diffuse into the cell [51–54]. LPS, composed of lipid A, core oligosaccharide and O-specific polysaccharide, is a primary contributor to the envelope permeability barrier [34]. LPS prevents entry into the cell of hydrophobic compounds, as well as hydrophilic compounds too large to pass through porin channels [1,35].

OM lipids and proteins are made at the IM or in the cytoplasm [55,56] and transported across the cell envelope driven by either by a concentration gradient [57] or energy-coupled transporters [58]. Envelope proteins are secreted by the IM ATP-dependent SecYEG translocon machinery [59]. After secretion, lipoproteins are lipidated and their signal sequence is cleaved before they are sorted to the IM or the OM [13,60]. OM-destined lipoproteins are trafficked to OM by the Lol (localization of lipoproteins) pathway [61–63]. Nascent OMPs are translocated across the periplasm by chaperone proteins [64–66] and assembled into the OM by a complex of proteins called the β-barrel assembly machine (Bam) [17,25,67–69]. Newly synthesized LPS molecules are transported and inserted into the OM via the seven-protein Lpt pathway [70–73].

Phospholipid transport is bidirectional, not energy-dependent [74,75], and maintains specific phospholipid composition differences between the two membranes [76–80]. A retrograde phospholipid transport pathway, Mla (maintenance of lipid asymmetry), has been well characterized, while anterograde phospholipid transport had largely remained a mystery until recently. In the Mla pathway, an OM lipoprotein, MlaA, removes mis-localized phospholipids from the outer leaflet of the OM, transferring them to the periplasmic protein MlaC, which returns the phospholipid to the IM through the ATPase MlaFEBD [16,81,82]. A dominant mutation in *mlaA* (mlaA*) results in increased OM permeability and LPS levels and cell death in stationary phase [83,84]. When the gene *yhdP* is deleted in this strain, anterograde phospholipid flow is significantly slowed [85]. This effect was somewhat mysterious until recent work suggested anterograde phospholipid flow is mediated by the IM proteins YhdP, TamB and YdbH [85–87]. Deletion of the three genes together is lethal [86,87], and lysis in a Δ*yhdP* Δ*tamB* mutant could be suppressed by loss of MlaA and PldA, an OM phospholipase [86]. Moreover, expressing *yhdP* alone from an inducible promoter resulted in a decrease in the amount of OM phospholipids compared to LPS [87], suggesting a role in phospholipid transport.

Interestingly, the effect of YhdP on the mlaA* mutant was not found to be shared by TamB and YdbH [85], and there are other unique phenotypes of the potential phospholipid transporters [25,88]. A study that examined the differences in phenotypes between these proteins identified another unique phenotype of Δ*yhdP*, a synthetic cold sensitivity with deletion of *fadR*, the gene for a transcriptional regulator modulating fatty acid metabolism [89]. The study provided evidence that the diversification of function between YhdP and TamB is related to phospholipid metabolism and suggests that the phospholipid transporters may have different transport specificities [89]. Thus, the importance of maintaining balanced synthesis of membranes across the Gram-negative cell envelope was demonstrated to be paramount. Given the ECA-dependent OM permeability found in a Δ*yhdP* mutant [25], the data from this study also suggest that ECA may play a role in maintaining that balance.

Although the focus of this review is ECA, Gram-negative bacteria have many cell surface polysaccharides that serve distinct functions to protect the cells against external stresses. O antigen is part of LPS and an important player in the membrane permeability barrier. Also very abundant on the cell surface are capsular exopolysaccharides, which form a covalently attached capsule around the cell [90,91] and protect the cell from host defences [92–94]. For example, colanic acid is a type of capsular exopolysaccharide that is associated with general responses to environmental stress conditions [95]. Capsular exopolysaccharides can also contribute to the formation of biofilm and protect bacteria from desiccation [96–99]. In addition to these, all Enterobacterales, except endosymbionts with highly reduced genomes [100,101], produce another carbohydrate antigen called ECA [38]. ECA is unique in that it is invariant in structure, unlike O antigen which is highly variable across different strains and species [102,103]. Due to this, identifying the function of this molecule has been of interest for many years.

## 3. Current understanding of enterobacterial common antigen function

The presence of ECA confers resistance to various external stressors in many Enterobacterales species and has been shown in several species and models to be important for pathogenesis. The effect of ECA on pathogenesis has been best studied in *Salmonella enterica* serovar Typhimurium (*S.* Typhimurium). Previous studies have shown that *S.* Typhimurium lacking ECA is more sensitive to bile salts and has reduced virulence compared to ECA-producing strains [104]. Specifically, mutations in the *wecD* and *wecA* genes of *S.* Typhimurium, two genes involved in the ECA biosynthesis pathway, cause sensitivity to the bile salt deoxycholate [104]. In the same study, competition assays showed wild-type strains of *S.* Typhimurium significantly outcompeted *wecD* and *wecA* mutants in both oral and intraperitoneal infections in mice [104], consistent with earlier studies [105]. Interestingly, since the pathogenesis defect was observed with intraperitoneal infection, which avoids bile salt contact in the gastrointestinal tract, this indicates that the effect of ECA is not simply due to bile salt resistance and that ECA represents an independent virulence factor. In addition to the well-studied effect of ECA on pathogenesis in *S.* Typhimurium, high-throughput screening studies have suggested ECA is important for urinary tract infection reoccurrence caused by uropathogenic *Escherichia coli* [106], pathogenesis of *Proteus mirabilis* during a polymicrobial urinary tract infection [107], and *Klebsiella pneumonia* bloodstream infection [108] in mice, and for *Enterobacter cloacae* pathogenesis in a wax worm model [109]. Taking these various studies into account, it seems likely that ECA plays an important role in survival in the host environment and in disease states caused by a broad range of Enterobacterales; however, further study into ECA’s role in pathogenicity is needed.

Beyond its role in pathogenesis, ECA has been shown to be important in acetic acid resistance [110], as well as resistance to antibiotics including nalidixic acid, gentamycin and vancomycin [111–113]. As mentioned above, a recent study on phospholipid transport suggested the effect of ECA on SDS EDTA (sodium dodecyl sulfate, a detergent and ethylenediaminetetraacetic acid, a divalent cation chelator, which prevents divalent cations from mediating bridging interactions between LPS molecules) and vancomycin resistance in a phospholipid transport mutant may relate to phospholipid metabolism [89]. The intriguing possibility that ECA’s other envelope permeability phenotypes may relate to the OM phospholipid composition will require further investigation. Another recent study focused on identifying genes important for *E. coli* growth during exposure to various aminoglycosides [114]. The authors used transposon-directed insertion sequencing (TraDIS) to identify genes important for fitness in the presence of either gentamicin, neomycin or streptomycin. The authors identified several genes which conferred increased fitness against multiple antibiotics, as well as fitness genes specific to either streptomycin, neomycin or gentamicin [114]. Interestingly, the fitness genes for neomycin resistance included those involved in ECA biosynthesis (encoded in the *wec* operon) and in magnesium sensing/transport (*phoPQ*) [114]. To validate their TraDIS results, the authors made single deletions to assay for growth defects; for neomycin, they made deletion mutants of the identified fitness genes *phoPQ*, *wecA, lpp* and *pal*. All the mutants displayed attenuated growth in the presence of neomycin [114]. As *wecA* is the first gene in the *wec* operon (figure 1) and is required for initiating the ECA biosynthesis pathway, the Δ*wecA* strain in this study having increased sensitivity to neomycin is a finding that agrees with previous studies that have shown ECA to be important for protection against other aminoglycosides like gentamycin and kanamycin [112,115].

ECA has also been shown to genetically interact with other systems involved in the envelope permeability barrier, including the Tol-Pal complex of proteins, which is involved in cell division and in maintaining OM lipid homeostasis in *E. coli* [116]. A recent paper [117] demonstrated that, in *E. coli*, strains deficient in the Tol-Pal complex have an accumulation of phospholipids in the OM and that a build up of ECA intermediates restores the defective OM barrier function, specifically against large hydrophilic molecules. However, the build up of intermediates does not restore the balance in OM lipid composition. So, although the exact function of these ECA intermediates remains unclear, the accumulation of ECA intermediates could be modifying envelope properties through other pathways not yet identified. Relatedly, in a recent preprint, Aoyagi *et al.* discovered a correlation between ECA and antimicrobial peptide resistance in rough *Yersinia pestis* strains [118]. However, the authors found that ECA itself does not play a role in enabling *Y. pestis* to infect its flea host *Xenopsylla cheopis*. Instead, ECA biosynthesis mutants such as Δ*wecE and* Δ*wecF*, which cause the build up of ECA intermediates and are predicted to decrease the availability of the lipid carrier used for synthesis of mean extracytoplasmic glycans (see §4), can lead to decreased flea colonization ability. This is likely due to cell envelope disruptions caused by sequestration of the isoprenoid carrier undecaprenyl phosphate (UndP, bactoprenyl), an essential 55-carbon long-chain isoprene, and substantial loss of aminoarabinose, resulting in increased antimicrobial peptide sensitivity [118]. These two recent papers highlight the highly complex interactions of the different cell envelope biosynthetic pathways, and how interpretation of high-throughput genetic screens can be confounded when it comes to ECA biosynthesis. Overall, although there have been significant advances in the understanding of ECA over the last six decades, its precise function in maintaining the permeability barrier remains elusive and will require further investigation.

## 4. Structure and biosynthesis of enterobacterial common antigen

ECA is a highly conserved carbohydrate moiety in Enterobacterales, which exists in three forms: two linear, surface-exposed forms and one cyclic form which is retained in the periplasm [40,41,119,120] (figure 1). All three forms consist of trisaccharide repeating units of *N*-acetyl-d-glucosamine (GlcNAc), *N*-acetyl-d-mannosaminuronic acid (ManNAcA) and 4-acetamido-4,6-dideoxy-d-galactose (Fuc4NAc) [121,122]. In *E. coli* K−12 grown at 37°C, these two linear forms possess a modal chain length of 6−7 repeating units [123]. The ECA chains are anchored to either the core oligosaccharide of LPS (ECA_LPS_) [41] or to diacylglycerol phosphate (ECA_PG_) [40]. Of the two surface-exposed linear ECA forms, ECA_PG_ is the dominant membrane-associated form and is present in all Enterobacterales, with increased levels of ECA_LPS_ observed in rough Enterobacterales that do not have O antigen [124]. The chain length of the cyclic form (ECA_CYC_) varies by species [119,125–127] but exists as four repeating units in *E. coli* K−12 [128].

The biosynthesis pathway for all three forms of ECA occurs on the inner side of the IM [129–133] and begins with WecA using UDP-GlcNAc as a substrate to attach GlcNAc-1-phosphate onto an isoprenoid carrier UndP to form Lipid I^ECA^ [134–136] (figure 1). Strains lacking the *wecA* gene do not produce any ECA [137,138]. After Lipid I^ECA^ is formed, WecB and WecC, a UDP-*N*-acetylglucosamine 2-epimerase and a UDP-*N*-acetyl-d-mannosamine dehydrogenase, respectively, catalyse reactions that result in the synthesis of UDP-ManNAcA from UDP-GlcNAc [129,139,140]. The WecG glycosyltransferase can then use UDP-ManNAcA to attach ManNAcA to lipid I^ECA^ to form lipid II^ECA^ [135,141] (figure 1). Recently, a series of bioinformatic analyses and immunoblotting assays were used to investigate the biochemistry of the WecG glycosyltransferase [142]. The authors determined that helix II of the C-terminal domain in *Shigella flexneri* is critical in keeping WecG peripherally associated with the cytoplasmic membrane [142]. They then identified three key hydrophobic residues in helix II that are likely responsible for facilitating this membrane association [142]. The authors also confirmed that WecG does not interact with WzyE, which then prompted them to investigate if lipid-mediated interactions between WecG and ECA lipid-I and/or lipid-II are responsible for peripherally retaining WecG to the membrane. Since no chemical treatments they applied were able to dissociate WecG from the whole membrane of the *wecC* mutant, the authors suggest that WecG is retained to the membrane through lipid interactions involving lipid I^ECA^, a finding that asserts a re-characterization of the protein as a peripherally associated membrane protein [142].

Finally, a series of reactions catalysed by the enzymes RffH (RmlA_ECA_), RffG (RmlB_ECA_), WecE and WecD convert glucose-1-phosphate into dTDP-Fuc4Nac, which is then by WecF used as a substrate to attach Fuc4NAc to lipid II^ECA^ and form lipid III^ECA^ [129,143,144] (figure 1). RffH and RffG have the same enzymatic activity as the O-antigen biosynthesis proteins, RmlA and RmlB, respectively, and so these proteins are partially redundant in their ECA synthesis role [143]. Lipid III^ECA^ must then be flipped from the cytoplasmic side of the IM to the periplasmic side; a process that is mediated by the WzxE flippase [145,146]. The structure of the *E. coli* WzxE flippase protein was very recently revealed via X-ray crystallography [147]. WzxE was determined to be composed of 12 transmembrane helices separated into two helical bundles at the transmembrane 7 (TM7), which rotates approximately 27˚ to facilitate the inward and outward-facing conformational states of the flippase [147]. Two pairs of conserved terminal arginine residues, one pair at the periplasmic end of the lumen and one at the cytoplasmic end, enable the protein’s flipping action. Whereas the cytoplasmic arginine pairs had a separation of 12 Å in the outward conformation and of 19 Å in the inward conformation, the periplasmic pair of arginines was separated by 19 Å in the outward confirmation but 12 Å in the inward [147]. The movement of these residues is proposed to function by enabling the passage of the pyrophosphate moiety of lipid III^ECA^ from the site of its initial recognition at the C-terminal arginine cluster of WzxE to the highly positively charged lumen where the pyrophosphate can then bind the periplasmic arginine pairs [147]. This binding triggers the rotation of TM7 and opening of the periplasmic face. These novel findings have illuminated the structure of the WzxE flippase and revealed potential insights into the complex interactions between the flippase and the ECA polymerase WzyE.

Once lipid III^ECA^ is on the periplasmic leaflet of the IM, the ECA chain is polymerized by WzyE, which works in conjunction with the chain-length regulator WzzE [123] (figure 1). Temperature has been shown to affect the modal chain length of membrane-bound ECA forms, where modal chain length is four repeating units at 30°C, but increases to 6−7 units at 37°C [39]. WzxE, WzyE and WzzE are all ECA-specific homologues of the Wzy-dependent pathway, a common polysaccharide biosynthetic pathway in Gram-negative bacteria most notably involved in O-antigen biosynthesis [148]. The interaction between the chain length modulator protein, WzzE and the polymerase, WzyE, was investigated by Leo *et al*. in 2021 using western blots and copurification experiments [149]. The authors used chimeric proteins that had one or both transmembrane regions of either WzzE or WzzB swapped. They found that when the WzzB transmembrane helix 2 was swapped into the WzzE protein, the strains expressing these chimeric proteins lost the ability to control the modal length of ECA and WzzE increased its interaction with WzyB [44]. These data suggest that transmembrane helix 2 of the Wzz proteins is important for interaction with the cognate Wzy polymerases to control chain length.

Additionally, a novel interaction between O antigen and ECA synthesis was recently discovered, whereby overexpressed WzzE can partially regulate O-antigen modal length via an interaction with WzyB, the O-antigen polymerase [149]. These findings demonstrated a level of ‘cross talk’ between the two polysaccharide synthesis systems. Relatedly, the first major study into the WzyE ECA polymerase of *S. flexneri* was conducted, which leveraged multiple sequence alignments and topology mapping [142]. This study showed that WzyE displays high peptide conservation across Enterobacterales and identified seven functionally conserved arginine residues within the periplasmic loops of WzyE, which appear to be important in maintaining the protein’s stability and function [142]. This is interesting to note since in the O-antigen polymerase, WzyB, protein sequences are highly variable [150–152]. However, it is perhaps logical that the structure of the ECA polymerase should be highly conserved when one considers the invariant nature of ECA compared with the diversity of O antigens.

In a 2023 study by Weckener *et al.*, high-resolution cryogenic electron microscopy (cryo-EM) was used to investigate the structure of the WzzE chain length regulator in *Pectobacterium atrosepticum* [153]. The authors confirmed a defined stoichiometry of 8 : 1, WzzE : WzyE proteins in this species. The authors also used Förster resonance energy transfer (FRET) to investigate the interactions between WzzE and the WzyE polymerase and confirmed that WzzE and WzyE are forming a complex to regulate chain length [153]. The authors therefore propose the following model for the Wzx/Wzy chain length regulation process: the nascent polymer begins to form inside the lumen and the interior of the periplasmic domain of WzzE provides some form of a molecular ruler, contrary to a previous hypothesis [154] that it was the outer surface of Wzz which acts as a ruler. The polymer continues to grow until it fills the periplasmic lumen in WzzE, which then destabilizes the Wzz octamer. Then once the ECA polymer is transferred to the next downstream step in the pathway, Wzz could re-assemble around Wzy and restart the polymerization cycle [153]. Importantly, this paper confirms the complexing of Wzy and Wzz proteins to regulate ECA chain length, a pairing that is common in other polymerization systems such as for LPS O antigen. Another paper utilized cryo-EM to show that WzzE possesses alternating up-down conformations of its L4 interdomain loops that make up the top of the periplasmic bell [155]. Here, the authors suggest that the alternating up-down loop organization of the loops indicates a likely ratchet-type mechanism for polysaccharide elongation.

Ultimately, the actions of the Wzy-dependent pathway proteins result in the synthesis of an ECA polymer of appropriate length. The polymer is then transferred from the isoprenoid carrier UndP and either cyclized and retained in the periplasm to form ECA_CYC_ or attached to a lipid and trafficked to the cell surface as either ECA_LPS_ or ECA_PG_. The remaining isoprenoid carrier is then released in a pyrophosphate form and dephosphorylated [156–158], before being flipped back to the cytoplasmic side of the IM, likely by DedA family proteins [159,160]. The isoprenoid carrier can then be reused for the assembly of new glycans. The exact mechanism of ECA_CYC_ cyclization has not yet been determined but is dependent on WzzE, since disrupting *wzzE* results in no ECA_CYC_ being produced [119,128,161]. There is also not yet a clear understanding of the genes and mechanisms involved in the synthesis and translocation of ECA_PG_. It is known that ECA_PG_ is synthesized by transferring the polysaccharide chain from the isoprenoid carrier to a lipid donor resulting in its linkage to diacylglycerol phosphate (DAG-P) through a phosphodiester bond. A recent paper investigated this reaction [162]. To determine the lipid donor that provides the DAG-P moiety to ECA_PG_, the authors conducted a series of overexpression and deletion assays of *E. coli* MG1655 K−12 phospholipid biosynthesis genes and discovered that alterations expected to increase levels of phosphatidylglycerol resulted in increased synthesis of ECA_PG_, and alterations expected to decrease levels of phosphatidylglycerol decreased ECA_PG_ synthesis [162]. This paper provided a crucial insight into the ECA biosynthesis pathway by demonstrating that ECA_PG_ can be synthesized in the absence of the other major phospholipids (PE and cardiolipin) and that phosphatidylglycerol is the lipid donor for the synthesis of ECA_PG_.

ECA_LPS_ is attached to the lipid A core oligosaccharide by the O-antigen ligase, WaaL [163], where it is then presumed to be transferred to the outer leaflet of the OM by the seven-protein trans-envelope pathway, Lpt [14,15,164,165], as are other forms of LPS. In 2020, a structural survey was conducted that evaluated the structure and linkage position of ECA_LPS_ to LPS core oligosaccharide in a variety of rough and smooth *E. coli* strains [166]. The authors evaluated purified ECA_LPS_-derived oligosaccharides using matrix-assisted laser desorption/ionization-time of flight (MALDI-TOF) mass spectrometry and NMR analysis to identify core oligosaccharide fractions substituted by at least one ECA repeating unit and to determine the exact linkage position to core oligosaccharide, respectively. It was previously assumed that the attachment site for the *E. coli* O antigen to the R4 core oligosaccharide was the side branch β-Galp, a glycosylgalactose [167], due to the high level of similarly between rough *E. coli* R1 and R4 WaaL proteins. This was therefore also the hypothesis for the site of ECA_LPS_ attachment. The MALDI-TOF and NMR spectra results from this paper confirmed that the ECA ligation site did fit the scheme of O-antigen/core oligosaccharide glycosidic linkage, but only for R1, R2 and R4 strains; no ECA_LPS_ was present in the rough R3 *E. coli* strain [166]. Importantly, another recent paper that sought to evaluate the frequency of heterogeneity in ECA structure present in supernatant after LPS ultracentrifugation determined that different ECA purification methods may yield more or less of certain ECA forms [168]. The authors in this paper observed high heterogeneity of supernatant-derived ECA that can contaminate LPS purified by ultracentrifugation [168]. This finding should be taken into consideration when evaluating studies involving purified ECA_LPS_.

Notably, the isoprenoid carrier, which acts as a scaffold for building ECA repeating units [161], is the same that is also used in the production of numerous other bacterial glycans including peptidoglycan and O antigen [169–171]. Therefore, certain disruptions of ECA synthesis have been hypothesized to impact the production of other polysaccharides by reducing the availability of the isoprenoid carrier due to its sequestration in biosynthetic intermediates [172,173]. A recent paper sought to determine whether the isoprenoid carrier used in ECA biosynthesis was decreased in mutants of the ECA glycosyltransferases and flippase (*wecA*, *wecG*, *wecF* and *wzxE*, respectively), as well as to directly assay the build up of ECA intermediates in these strains [174]. The authors found that cells lacking *wecA* had an almost twofold increase in isoprenoid carrier quantity, suggesting that when the isoprenoid carrier is not being used to produce ECA, it is more available for use in other pathways [174]. However, the authors also found that *ΔwecG* and *ΔwecF* mutants resulted in significant increases in isoprenoid carrier levels, despite an accumulation of lipid I^ECA^ and lipid II^ECA^ [42]. In contrast, the *ΔwzxE* mutant resulted in a twofold decrease in isoprenoid carrier compared to wild-type, indicating that isoprenoid carrier sequestration is greater in the *wzxE* mutation compared to the glycosyltransferases. These findings are interesting because it had been previously shown that cells with the ECA flippase *wzxE* or aminotransferase *wecE* deleted exhibited significant morphological abnormalities including cell swelling and filamentation [173], while cells deleted for wecF and wecG also showed cell shape defects as well but were less severe than the *wecE* and *wzxE* mutants [173]. Therefore, the actual levels of isoprenoid carrier sequestration do not fully correlate with the peptidoglycan and cell shape defects observed in ECA biosynthesis mutants. This study highlights the many complex interdependencies between the various glycan synthesis pathways. Taken together, this and other recent studies have contributed greatly to our increased understanding of the mechanisms involved in synthesizing ECA; however, much remains to be discovered regarding the regulation of ECA biosynthesis.

## 5. Regulation of cell surface carbohydrates

Biosynthetic processes for most surface-exposed carbohydrates begin with the isoprenoid carrier, UndP. Much interdependence exists between these different cell-wall biosynthetic pathways since they use many of the same substrate precursors as well as the same isoprenoid carrier. In fact, in 2022 Maczuga *et al.* demonstrated in *S. flexneri* that mutations made in either the ECA biosynthesis pathway or the O-antigen pathway can affect each other, depending on where the mutation occurred in the synthesis pathway [142]. Previous work had demonstrated that mutations in either of these pathways separately affects peptidoglycan synthesis [173,175] (see [176] for a review of peptidoglycan biosynthesis). In the 2022 study of Maczuga *et al.*, they used a variety of *wzy* and *wec* mutants to investigate ECA and O antigen levels using ECA western immunoblots and silver staining and also observed for phenotypic changes using phase-contrast microscopy [142]. Through these methods, the authors saw that when the *wzyB*/*wzyE* homologues were deleted, there was an associated reduction in the amount of reciprocal OM polysaccharide, ECA or O antigen [142]. This suggested that disruption of either the ECA or O-antigen biosynthesis pathways was interfering with the other, most likely due to isoprenoid carrier sequestration. This interdependence has also been highlighted by a study that has found that cell survival after disruption of O-antigen synthesis in *S. flexneri* and O-antigen-producing *E. coli* K−12 depends on the activity of the ECA synthesis pathway [177]. The authors propose that the isoprenoid carrier pool is maintained by modulating the allocation of the isoprenoid carrier between the biosynthesis pathways.

O antigens are highly variable in the order and composition of their polysaccharide [178–182]. O-antigen biosynthesis genes are generally contained in biosynthetic gene clusters [130,183–186] and the variety of O antigens reflect genetic variation in these clusters. O-antigens are subject to intense selection by the host immune system, bacteriophages and other environmental factors [187], accounting for the variety of O-antigens that exist within even one species [188]. For example, several bacteria, including *S.* Typhimurium*,* possess two *wzz* genes, which allow for the bimodal distribution of modal chain length [189,190]; the regulation of which may be related to serum sensitivity [189]. In *S.* Typhimurium, the transcriptional anti-terminator RfaH regulates O-antigen biosynthesis [191,192], and the expression of RfaH is regulated by RpoN [191]. This regulation varies based on growth phase and environmental conditions, with high levels of expression in stationary phase [193] and under conditions of nitrogen limitation [191].

The synthesis and regulation capsular polysaccharides (a.k.a. K antigens) have been recently reviewed in depth [194]; however, a few examples are provided herein for context. The K antigens of *E. coli* have been separated into four different ‘groups’ based on their biochemical and genetic properties. Capsular polysaccharides are synthesized by gene clusters [90] and the regulation of expression varies by group. For example, Group 1 capsule clusters are distinguished by their regulation by the Rcs (regulation of capsule synthesis) phosphorelay stress response system [195–197]. Conversely, Group 2 capsules are regulated by temperature, being optimally expressed at 37°C [198,199]. Several different regulators of Group 2 antigens are involved at different growth phases [200]. Group 4 K-antigens are also known as ‘O-antigen capsules’ due to their structural similarity to the O antigen of LPS [201,202]. In *Salmonella enteritidis*, Group 4 capsules are regulated by the protein AgfD and are expressed maximally in stationary phase at temperatures below 30°C [203]. Group 3 capsular polysaccharides are now considered a subgroup of the classical Group 2 capsules, and no experimentally based insights have been made into the regulation of these capsules [194].

Although not structurally or functionally related to carbohydrate antigens, flagella are one of the classic cell surface antigens (H-antigen). The expression of flagellar biosynthesis genes is very tightly regulated and primarily controlled by the transcriptional regulatory proteins FliDC, FliA and FlgM; however, flagellar biosynthesis is also regulated by other regulatory proteins such as RcsB and IHF, small RNAs (sRNAs), and by various environmental signals [204]. For example, in EHEC O157:H7, environmental factors that affect expression of flagellar genes include presence of SCFAs, mucin, bile salts, epinephrine/norepinephrine and indole [205–211].

## 6. Regulation of enterobacterial common antigen biosynthesis

More similar in structure to O and K antigens, ECA is an invariant carbohydrate retained on the cell surface of Enterobacterales. In recent years, several key insights have been made that help unravel the complex regulation pathways and elements involved in ECA synthesis. While investigating ECA as possible receptor for the podovirus N4 bacteriophage [212,213], recent work made some key discoveries relating to *wec* operon expression [214]. Since mutations in the acetylglucosamine epimerase *wecB* (*nfrC*) had previously been shown to confer resistance to N4, the authors conducted a challenge with N4 phage in strains lacking *wecA* (the first gene in the ECA biosynthesis operon), *wecB*, or lacking the entire *wec* operon (Δ*eca*) [214]. They found that only deletions of *wecB* and Δ*eca* resulted in resistance to N4, but the Δ*wecA* strain did not [214]. This indicated ECA was not the primary receptor for N4 phage, but that it is instead a novel glycan that needs WecB for its synthesis. Importantly, given the reliance on WecB for its infectivity, N4 resistance can still be used as a proxy for *wec* operon expression.

**Figure 2.**
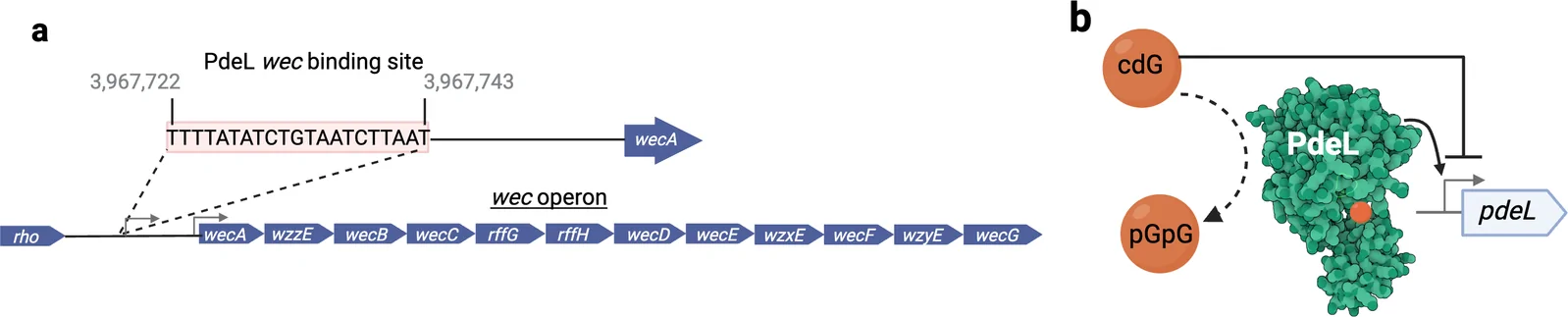
Function and regulation of the cyclic di-GMP phosphodiesterase PdeL. (a) The *wec* promoter binding site sequence of PdeL, as determined by [214], in the context of the *wec* operon in *E. coli* K 12. (b) PdeL has an autoregulatory function in addition to its function as a transcriptional regulator of genes related to motility, N4 bacteriophage resistance, and ECA biosynthesis and its transcription is also negatively regulated by cdG. Dashed arrow indicates enzymatic regulation of cdG by PdeL. Solid arrow indicates autoregulation of *pdeL* transcription [215]. Created in BioRender (Bennett, H. (2025) https://BioRender.com/k62c544)

The authors wanted to determine how these bacteria maintain the expression of an invariant surface glycan, despite phage predation. In the course of their investigation, they identified a partially N4-resistant mutant that contained an insertion sequence that results in the constitutive expression of the phosphodiesterase gene *pdeH*, resulting in significantly reduced cyclic di-GMP levels (cdG) [214]. Unlike the incomplete N4 phage protection from increased *pdeH* expression, expression of another cyclic di-GMP phosphodiesterase and transcriptional regulator, PdeL, provided complete protection [214]. Combined, these data indicate that cyclic di-GMP is indeed a requirement for N4 phage infection and that PdeL, but not PdeH, results in complete protection against N4 phage [214]. Intrigued by this finding, the authors conducted ChIP-Seq to determine if PdeL was transcriptionally regulating genes involved in N4 phage infection in addition to modulating cdG levels. They discovered eight novel binding sites for PdeL, one of which mapped to the promoter region of the *wec* operon. Surface plasmon resonance (SPR) using short overlapping fragments of the *wec* promoter region identified the exact binding site of this novel *wec* regulator. Importantly, PdeL is the first experimentally confirmed transcriptional regulator of the *wec* operon (figure 2). Besides PdeL, one other potential transcriptional regulator of the *wec* operon, NsrR, has been identified via ChIP-Seq as having a binding site in the promoter region but its regulatory activity has not yet been confirmed [216].

In addition to transcriptional regulation, ECA biosynthesis regulation has been shown to occur at the post-transcriptional level. A paper was published that investigated the process by which the DAG-P-linked form of ECA, ECA_PG_, is produced from polymerized ECA [217]. The authors used TraDIS [218] in *E. coli* K−12 to conduct a screen for genes that were essential in a strain making ECA_PG_ but not in strains making all forms of ECA or no ECA in an effort to identify genes potentially involved in ECA_PG_ biosynthesis [217]. They hypothesized that these genes would be essential in an ECA_PG_ only strain due to sequestration of the isoprenoid carrier from peptidoglycan synthesis. They identified *elyC,* the gene for a protein previously been shown to alter PG biosynthesis rates, particularly at lower temperatures [219], as essential specifically in the ECA_PG_ only strain [217]. Deletions of *elyC* resulted in greatly increased ECA_PG_ levels, while ElyC had less of an effect on ECA_PG_ levels in strains lacking WzzE and thereby ECA_CYC_. Additionally, in strains lacking ECA_LPS_ the ECA polymers that would have normally been applied to making ECA_LPS_ are reallocated to between the two remaining forms, but only ECA_PG_ levels decreased while ECA_CYC_ levels remained static [217]. Together, these results suggest that ECA_CYC_ levels are monitored by ElyC to regulate ECA_PG_ production and overall ECA levels (figure 1). Transcriptional reporter assays of *elyC* overexpression strains and *elyC* deletion strains yielded no changes in *wec* operon promoter activity [217]. All these data combined suggest a novel pathway where ElyC provides feedback regulation of ECA_PG_ production based on the periplasmic levels of ECA_CYC_.

Several environmental conditions have also been shown to result in changes to transcription of *wec* genes. For example, in 2008 Jarboe *et al.* conducted a series of experiments to identify the regulatory response elements activated in *E. coli* under conditions of varying levels of S-nitrosoglutathione*,* an endogenous source of bioavailable nitric oxide (NO) [220]. They employed transcriptomic analysis and the use of a bioinformatic tool called network component analysis (NCA) to deduce transcriptional networks from their real-time qPCR, growth curve, and other experimental data. The authors found that depleting cysteine and homocysteine, the targets of GSNO, results in many downstream effects including growth inhibition and altered activity of the transcription factors CysB, MetJ and MetR, which subsequently causes disruption in the methionine biosynthesis pathway [220]. Most relevant to this discussion, the authors here also found via their microarray data that *wecA*(*rfe*) transcription is downregulated in a statistically significant manner by increased levels of S-nitrosoglutathione [220]. A summary of currently known ECA regulatory factors is contained in table 1. One final consideration regarding ECA regulation is that a previous study from 2005 had demonstrated that ECA_CYC_ synthesis occurred throughout the exponential phase and for a prolonged period into exponential phase [128]. This suggests that at least ECA_CYC_ may be important during different growth phases and presumably different regulatory factors may be at play during these different growth phases. Further investigation into the regulation of ECA’s biosynthesis will provide insights into the environmental conditions where ECA is important.

**Table 1.** Regulatory influences on ECA biosynthesis.

regulatory factor	level of regulation	direction of regulation	type of ECA affected	references
PdeL	transcriptional	repressor	*wec* operon	[214]
NsrR	transcriptional *(not yet experimentally confirmed)*	repressor	*wec* operon	[216]
ElyC (in conjunction with ECA_CYC_)	post-transcriptional	negative feedback loop	ECA_PG_	[217]
S-nitrosoglutathione	transcriptional	repression	*wec* operon	[220]

## 7. Concluding remarks

Since its discovery in the 1960s, a multitude of researchers in the field have made substantial contributions to our understanding of ECA. Over the last six decades, numerous papers have been published on ECA, enabling significant advancements in our understanding of its structure, function, regulation and involvement in conferring resistance to a number of stressors. However, a precise function for ECA remains elusive and continued work is necessary to thoroughly characterize the function and molecular mechanisms of ECA’s protective capabilities. An interesting observation based on recent data is the variation in expression levels and regulation across the three forms when exposed to different antibiotics, toxic compounds, levels of certain intracellular molecules and in different genetic backgrounds or environmental conditions all suggest the functions of each form may also be different. Continued efforts focused on studying the intracellular systems, regulatory elements and environmental conditions important in coordinating expression levels of each of the three forms of ECA will be crucial avenues of investigation to further characterize ECA’s function and identify targets for future therapeutics against resistant infections.

## Data Availability

This article has no additional data.
